# *Piezo* Is Essential for Amiloride-Sensitive Stretch-Activated Mechanotransduction in Larval *Drosophila* Dorsal Bipolar Dendritic Sensory Neurons

**DOI:** 10.1371/journal.pone.0130969

**Published:** 2015-07-17

**Authors:** Thomas J. Suslak, Sonia Watson, Karen J. Thompson, Fiona C. Shenton, Guy S. Bewick, J. Douglas Armstrong, Andrew P. Jarman

**Affiliations:** 1 Doctoral Training Centre in Neuroinformatics and Neural Computation, University of Edinburgh, Edinburgh, United Kingdom; 2 Centre for Integrative Physiology, University of Edinburgh, Edinburgh, United Kingdom; 3 Institute for Adaptive and Neural Computation, University of Edinburgh, Edinburgh, United Kingdom; 4 Institute of Medical Sciences, University of Aberdeen, Aberdeen, United Kingdom; 5 Institute for Ageing, University of Newcastle, Newcastle, United Kingdom; Columbia University, UNITED STATES

## Abstract

Stretch-activated afferent neurons, such as those of mammalian muscle spindles, are essential for proprioception and motor co-ordination, but the underlying mechanisms of mechanotransduction are poorly understood. The dorsal bipolar dendritic (*dbd*) sensory neurons are putative stretch receptors in the *Drosophila* larval body wall. We have developed an *in vivo* protocol to obtain receptor potential recordings from intact *dbd* neurons in response to stretch. Receptor potential changes in *dbd* neurons in response to stretch showed a complex, dynamic profile with similar characteristics to those previously observed for mammalian muscle spindles. These profiles were reproduced by a general *in silico* model of stretch-activated neurons. This *in silico* model predicts an essential role for a mechanosensory cation channel (MSC) in all aspects of receptor potential generation. Using pharmacological and genetic techniques, we identified the mechanosensory channel, DmPiezo, in this functional role in *dbd* neurons, with TRPA1 playing a subsidiary role. We also show that rat muscle spindles exhibit a ruthenium red-sensitive current, but found no expression evidence to suggest that this corresponds to Piezo activity. In summary, we show that the *dbd* neuron is a stretch receptor and demonstrate that this neuron is a tractable model for investigating mechanisms of mechanotransduction.

## Introduction

Mechanotransduction is key to essential sensory functions, such as touch and proprioception. Organisms typically possess many types of mechanosensory endings [1], and in mammals, detection of changes in muscle length is mediated via muscle spindles [reviewed in 2]. A similar role is played by muscle stretch receptors in arthropods, such as the stretch receptor organs (SRO) of insects [3, 4]. The SRO of the *Manduca sexta* larva plays a role in providing feedback on tension within each body segment during the peristaltic segmental contractions of locomotion [5]. In the *Drosophila* larva, the dorsal bipolar dendritic (*dbd*) sensory neuron, whose two dendrites span the segment in parallel to the major horizontal muscles, may perform a similar function, but this has not been established [6].

Functional similarities between stretch receptors may suggest underlying mechanistic similarities. Indeed, crayfish muscle stretch receptors and vertebrate muscle spindles show strikingly similar electrophysiological responses to stretch stimuli [7, 8, 9]. Furthermore, these responses can be described and reproduced in biophysical models [10, 11], which potentially provide predictive tools for identifying the proteins of the mechanosensory response. Despite the wealth of information on the structure, pharmacology and electrophysiology of vertebrate muscle spindles, relatively little is known of the mechanosensitive molecular components involved in transduction [12].

The genetic tractability of *Drosophila* and the accessibility of its *dbd* neurons potentially provide a route to understanding the molecular mechanisms of stretch receptor mechanotransduction. To this end, we have investigated whether *dbd* neurons are amenable to patch-clamp electrophysiology. Here, we report the stretch-evoked receptor potential profiles of *dbd* neurons *in situ* and compare the responses recorded to those of mammalian muscle spindles, which demonstrate that they are indeed stretch receptors. Combining electrophysiological recordings of the receptor potential with genetics, pharmacology and mathematical modelling, we then examine the mechanosensory mechanism, and in particular the role of two candidate mechanotransduction proteins known to be expressed in *dbd* neurons: TRPA1 and DmPiezo [13, 14].

## Materials and Methods

### Fly lines

Experiments use *w* ^*1118*^, a *Piezo-Gal4* driver strain (donated by S.E. Kim), *P{KK101815}VIE-260B*, *w* ^*1118*^ *; P{GD993}v2796*, *w* ^*1118*^ *; P{GD2375}v37249* (Vienna *Drosophila* Resource Center) and *w* ^*1118*^ *; TrpA1* ^*1*^, (Bloomington *Drosophila* Stock Center). Fluorescence imaging used yw; Gal4^109-80^, UAS-mCD3-GFP.

### Fly dissection

Third instar larvae were pinned rostrally and caudally in a 35mm Sylgard-lined dish containing the standard *Drosophila* electrophysiological recording solution, HL3 (70mM NaCl, 5mM KCl, 1.5mM CaCl_2_, 20mM MgCl_2_, 10mM NaHCO_3_, 5mM trehalose, 115mM sucrose, 5mM HEPES, pH 7.4 [15]). One longitudinal lateral incision was made followed by rostral and caudal transverse incisions of the superior pelt and evisceration. The dissected pelt was opened to expose the innermost aspect and pinned four-square.

### Fly electrophysiology

Dissected larvae were viewed at 400x magnification. A small portion of muscle overlying the *dbd* neuron was digested with 1% Type-XIV protease [Sigma], administered via a large-diameter patch electrode. Recordings were made in whole-cell configuration (electrode internal solution: 140mM KCH_3_SO_3_, 2mM MgCl_2_, 2mM EGTA, 5mM KCl, 20mM HEPES [16]), and recorded to computer hard drive using WinWCP [University of Strathclyde]. Ramp-and-hold stretch protocols were applied to the pin through the head of the dissected preparation, stretching the intact body wall muscles and thus the *dbd* neuron. Larval pelt stretches of 76μm, 84μm and 92μm, corresponding to 0.5nA, 1nA and 2nA current pulses, were generated in WinWCP, to drive a piezoelectric wafer (PZT507, Morgan Electro Ceramics, UK). A fire-polished probe of borosilicate micropipette glass was affixed to the wafer to mechanically stimulate the pin at the head of the preparation, which in turn stretched the *dbd* neurons, *in vivo*.

### Fly immunohistochemistry

Dissected pelts were fixed overnight at 4°C in 1% w/v formaldehyde, made up in HL3. Fixed pelts were washed 3 times, at room temperature, in PAT3 (0.5% Bovine serum albumin and 0.5% Triton X-100 in standard phosphate buffered saline [17]), for one hour each, then in blocking buffer for 2 hours at room temperature. Primary antibody (1/1000 rabbit α-GFP) was applied at room temperature for 2 hours, then moved to 4°C overnight. The preparation was again washed 3 times, for one hour each time, with PAT3, followed by incubation with secondary antibody (1/400 GFP goat α-rabbit) for 2 hours at room temperature, then 5 days at 4°C. Three final PAT3 washes were performed, as before, followed by 1 PBS wash. Preparations were slide-mounted in Vectashield.

### Rat dissection

Tissue harvest, preparation and recording techniques were as previously published [18], and are only described here, briefly. Adult male Sprague-Dawley rats of 389-410g were killed by CO_2_ overdose, in accordance with Schedule 1 of the U.K. and E.U. ethical legislation of the Animals (Scientific Procedures) Act, 1986 and 63/2010/EU.

### Rat spindle electrophysiology

Hind paws were removed, skinned and placed in gassed (95% O_2_/ 5% CO_2_) Liley’s saline (138.8mM NaCl, 4mM KCl, 12mM NaHCO_3_, 1mM KH_2_PO_4_, 1mM MgCl_2_, 2mM CaCl_2_ and 11mM glucose [19]). The 4^th^ deep lumbrical nerve-muscle preparations of both paws were dissected out and pinned in silicon rubber-lined tissue culture dishes under Liley’s saline. Salts were obtained from Sigma-Aldrich [Dorset, England, UK] or Fisher Scientific [Loughborough, England, UK]. All experiments were carried out at room temperature (18–21°C). Following 40 minutes in gassed saline to equilibrate, the bone was securely pinned. The tendon was hooked to a three-axis micromanipulator [Narishige, Japan]. Electroneurograms were recorded *en passant* from the muscle nerve using silver-wire electrodes. Signals were amplified [A103, Isleworth Electronics, Isleworth, UK and 8102, CF Palmer, High Wycombe UK pre-amplifiers in series], and displayed and recorded simultaneously on WinWCP [University of Strathclyde]. The minimum firing length was found at the beginning of each experiment (the muscle length at which minimal firing is seen). The muscle was stretched by 1mm for 5s before returning the muscle to its original length for a further 5s. This “stretch-and-hold” procedure was repeated 3 times. In all experiments, three consistent control recordings were taken prior to compound addition to obtain a baseline. Ruthenium red was added for an hour before recording three further stretch-and-hold cycles, followed by a saline wash.

### Western blotting

#### Spindle dissection

The head was removed and skinned. The lateral portion of each deep masseter muscle was removed and used as a negative control, as it contains no muscle spindles [20]. The rostro-medial portion of each deep masseter muscle was divided into three 2mm width longitudinal strips and lightly dissociated with 0.1% Type I collagenase [Sigma, UK] diluted in gassed (95% O_2_, 5% CO_2_) physiological saline [19] for 15 minutes at 37°C. Preparations were then stained with 0.05% methylene blue diluted in Liley’s saline with the addition of protease inhibitors to block further dissociation [0.5x Complete, Mini Protease Inhibitor Cocktail, Roche, UK] for 40 minutes at 37°C. Preparations were washed 4x 15 minutes in Liley’s saline at 37°C. Individual muscle spindles, visualised with methylene blue, were then removed by microdissection, snap-frozen on dry ice and stored at -80°C until use. *SDS-PAGE*: Muscle spindle samples were pooled from 5 separate rats to give approximately 1000 nerve endings per sample. Protein was extracted from both lung and muscle spindle samples using 150mM NaCl, 50mM HEPES, pH7.3, 1% Triton with the protease inhibitor cocktail. Protein concentrations in each sample were quantified by BCA assay [Pierce, UK]. Samples were loaded with Laemmli buffer [21] at a concentration of 10μg/μl, and run on an 8% polyacrylamide gel at 150V for 75 minutes. *Immunoblotting*: After SDS-PAGE, gels were transferred onto nitrocellulose membrane at 100V for 90 minutes in a tris-glycine-methanol buffer. Membranes were washed three times in Tris buffered saline with 0.5% Tween (TBST), then placed in blocking buffer—TBST +5% dried skimmed milk powder—for 1 hour. Membranes were incubated overnight using the following antibodies: Piezo1 [1:500; Santa-Cruz (Insight Biotechnology)], Piezo2 [1:1000; Sigma, UK and 1:1000; Abcam, UK (Fam38B)]. These antibodies were not validated beyond that performed by the manufacturers. All antibodies were diluted in TBST + 5% dried skimmed milk powder. Membranes were washed three times in TBST. Membranes were either incubated in HRP-conjugated goat polyclonal α-rabbit secondary antibody [Piezo2, Fam38B; Abcam; 1:1000] or HRP-conjugated α-goat secondary antibody [1:5000; Sigma, UK]. Membranes were developed by enhanced chemiluminescence method and imaged using a FluorChem FC2 MultiImage II imager [Alpha Innotech, UK]. Images were captured using AlphaView software. Following imaging, membranes were reprobed with GAPDH antibody [Abcam, UK] to ensure protein loading per gel lane was equal.

### Rat Immunohistochemistry

#### Sections

Medial (spindle-containing) portions of deep masseter muscles were sectioned at 10μm thickness on a Leica 1850 UV cryostat, and mounted on Superfrost polylysine coated slides [Fisherbrand, UK]. Slides were stored at -80°C until use. Sections were air dried for 1 hour then fixed in acetone at room temperature for 45 minutes. Sections were washed 3 times for 5 minutes each in PBS then placed in blocking buffer (40ml PBS, 400μl Triton-X100, 0.8g BSA) for one hour to block non-specific binding. Sections were then incubated in primary antibody at 5°C overnight in either α-Piezo2 [1:500; Sigma, UK] or α-Fam38B [1:1000; Abcam, UK], and double-labelled with α-GAPDH [1:500; Abcam, UK] to identify spindle endings. Sections were then washed 3 times in PBS for 5 minutes each and incubated in fluorescent secondary antibodies [AlexaFluor goat α-Rabbit 594 and AlexaFluor donkey α-Mouse 350; Invitrogen, UK] at 5°C for 1 hour and imaged using a Nikon Eclipse E400 microscope, and images were captured using Volocity software [Perkin Elmer, UK].

#### Whole mounts

Deep lumbrical muscles were fixed in 4% formaldehyde in 0.1 m sodium phosphate buffer, pH 7.4, teased and squashed between a pair of microscope slides to facilitate penetration of the antibodies. Muscles were washed in buffer and blocked (1 h at room temperature) in PBS (pH 7.4) containing 0.25% BSA, 0.3% Triton X-100 and 0.02% sodium azide. The blocking buffer was also used as the antibody diluent. Preparations were double-stained with mouse monoclonal anti-synaptophysin [MAB5258, Millipore, UK] to label the glutamatergic vesicles inside the sensory terminals [18] and rabbit polyclonal anti-TRPC1 [sc20110, Santa Cruz, CA, USA]. Secondary incubation used Alexa Fluor (AF)-conjugated antibodies [AF 488 goat α-mouse IgG and AF 594 goat α-rabbit IgG respectively from Invitrogen, Eugene, OR, USA] followed by 3 washes in PBS. Muscles were mounted in glycerol–PBS [Citifluor AF1, Agar Scientific, UK] for confocal microscopy [MicroRadiance, Biorad, UK].

### Modelling

Modelling *in silico* was performed in Matlab. The model is developed from a previous biophysical stretch receptor model [11], which proposed a probabilistic mechanism of mechanosensory and voltage-gated ion channel coactivation that can reproduce the recordings of receptor potentials. A brief description of this previous model follows. The relationship of receptor tension to mechanical extension is modelled as a non-linear and linear spring in series (i.e. comprising both elastic and inelastic receptor components). This tensile force determines the open probability of a hypothesised mechanosensory cation channel, based on parameters derived from crayfish stretch receptor studies [10]. Further components in the model represent the open probabilities and leak conductances associated with hypothesised voltage-gated sodium and potassium channels (VNaC and VKC) [11]. The details of the changes to parameters of this previous model are described in the relevant section of the Results. Source code is freely available on GitHub, through Open Source Brain (http://www.opensourcebrain.org/ projects/dbdflymodel).

### Statistical analyses

Statistical significance was assessed in all comparisons using One-way ANOVA with Tukey post-hoc correction (fly data) or Bonferroni post-hoc correction (muscle spindle data). Throughout, *n* refers to the number of animals (fly data) or number of muscles (muscle spindle data).

## Results

### Characterising *dbd* neuron responses to stretch stimuli

**Fig 1:**
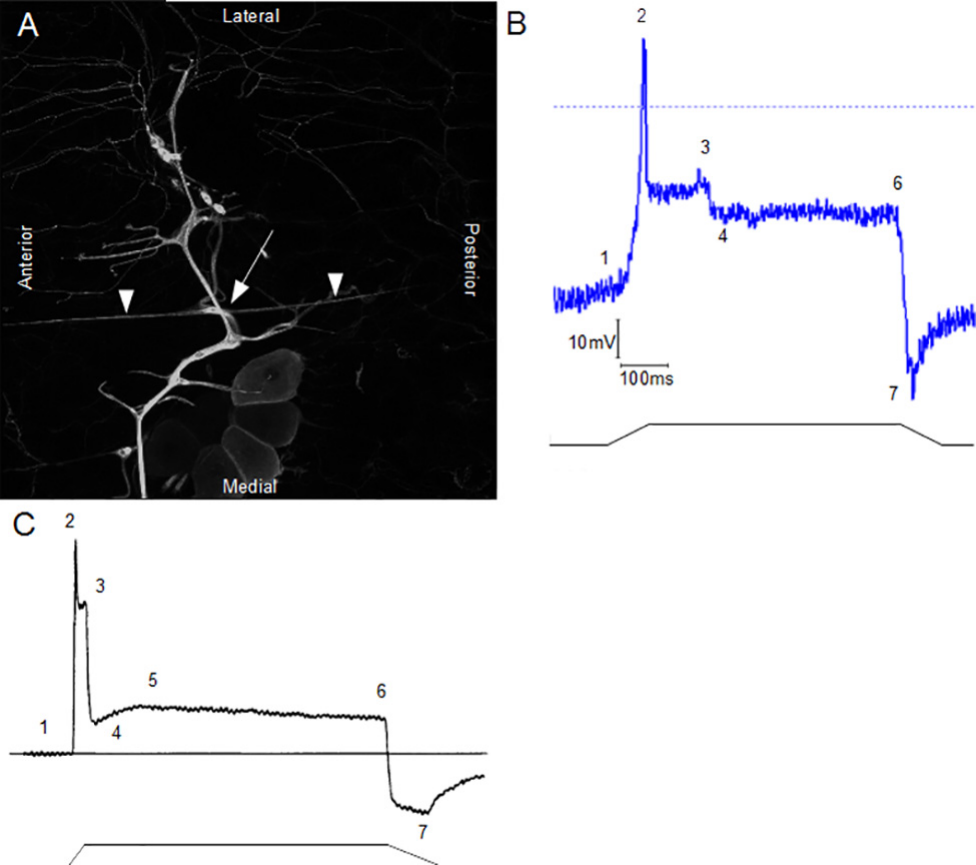
Stretch-activated features of *dbd* neuron receptor potentials are similar to those in muscle spindles. (A) Immunostaining of yw; Gal4^109-80^, UAS-mCD3-GFP larval thoracic segment PNS (peripheral nervous system) with α-GFP. The *dbd* neuron is readily identifiable by its distinctive morphology–a cell body (arrow), present in each thoraco-abdominal segment, with two long dendrites (arrowheads) projecting longitudinally along the anterior/posterior axis. These dendrites are thought to be the sensory transduction elements. (B) Stretch-evoked receptor potential recording from *dbd* neurons exhibit a complex receptor potential profile strongly resembling that in mammalian muscle spindles (sample trace from one such recording shown) (C). The numbers indicate common features with the mammalian potential. The corresponding ramp-and-hold stretch stimulus is indicated by the black line. (C) Distinctive receptor potential profile of stretch-activated mammalian muscle spindle. During dynamic stretching, there is a large initial depolarisation (1–2, defined here as dEm), followed by a partial repolarisation (3). Upon transitioning to static stretch, the receptor repolarises to a stable hold potential (4–6), which is maintained throughout static stretch. Following release, the receptor hyperpolarises (7), and returns to resting membrane potential. Again, the corresponding ramp-and-hold stimulus profile is indicated by the lower line [modified from 7]. Most of these features are present in the *dbd* neuron receptor potentials (B).

Previous studies have achieved whole-cell patch recordings from larval *Drosophila* neurons [22] including the *dbd* neuron [16]. However, they have not attempted mechanical stimulation of putative mechanosensitive endings in this configuration. We prepared filleted larvae and patched *dbd* neurons (Fig 1A) in whole-cell configuration. Stretch stimuli were applied to the pin in the head of each preparation, elongating the larva, thus applying stretch stimuli to the patched *dbd* neuron. By mechanically stimulating the cell, we were able to obtain recordings of the *dbd* neuron receptor potential generated in response to a stretch stimulus. Despite the simplicity of the ramp and hold stimulus, the overall profile of the receptor potential is complex (Fig 1B), with several distinguishable features—an initial depolarisation is followed by a lower hold potential; the end of the stimulus results in transient hyperpolarisation. Strikingly, most of these same features had also been observed for mammalian muscle spindles (Fig 1C) [7]. Consequently, we then performed quantitative comparisons of features of the *dbd* receptor potential profiles with those reported for mammalian muscle spindles.

**Fig 2:**
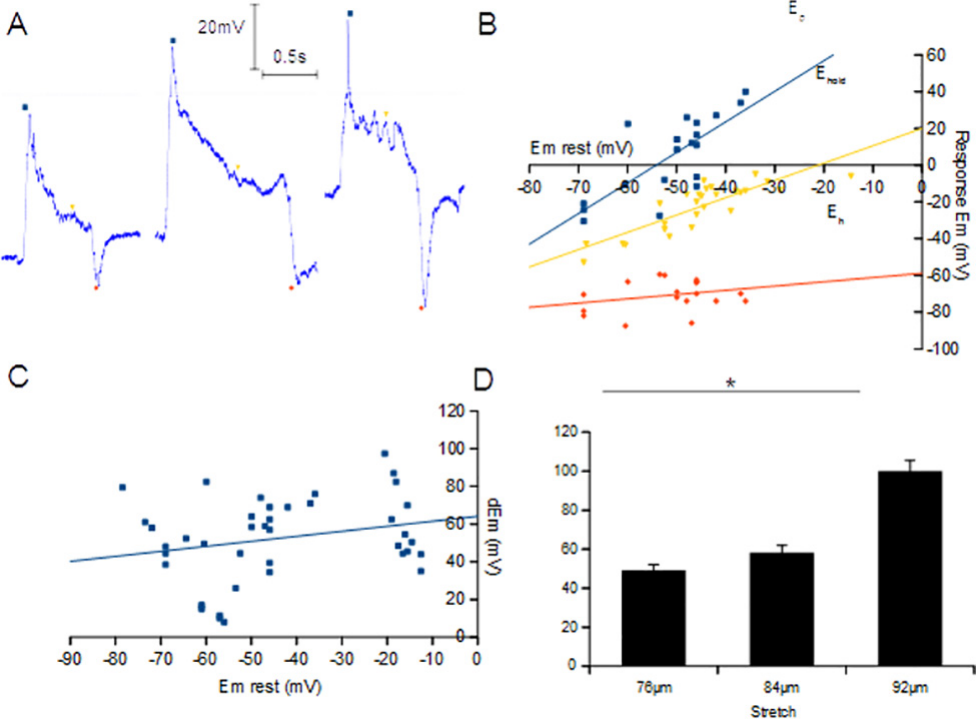
Key *dbd* receptor potential relationships resemble those of mammalian muscle spindles. (A) Receptor potential recordings from intact *dbd* neuron preparations vary in their initial resting membrane potential (*E*_*mrest*_), reflecting differing basal levels of pelt stretch prior to stimulation (sample traces from typical recordings at each level of pelt stretch shown). The three most distinctive features of the stretch-evoked response profile to a standard mechanical stimulus were analysed for their relationship to this difference in initial *E*_*mrest*_ in a range of such *dbd* neurons: peak depolarisation (*E*_*p*_; blue square, corresponding to 2 in Fig 1B), *E*_*hold*_ (yellow inverted triangle, 4–6 in Fig 1B) and *E*_*h*_ (orange diamond, 7 in Fig 1B). (B) The maximum amplitude of *E*_*p*_ and *E*_*hold*_ varied directly in proportion to the pre-stimulus potential, whilst *E*_*h*_ was relatively independent of *E*_*mrest*_ (*n* = 10). (C) Whilst the absolute voltage of *E*_*p*_ significantly varies in proportion to the pre-stimulus potential (Pearson correlation = 0.76, *n* = 10), the relative change in depolarisation amplitude from baseline (*dEm* = *E*_*p*_-*E*_*mrest*_, see also Fig 1) shows only a weak relationship to *E*_*mrest*_ in *dbd* neurons (Pearson correlation = 0.42, *n =* 10), consistent with previous findings in muscle spindles [7]. (D) *E*_*p*_ also shows a high degree of direct correlation with the amplitude of mechanical displacement (*p*<0.0001, *n* = 5).

Quantitative analyses were made by investigating the relationship between differing pre-stretch resting membrane potentials (*E*_*mrest*_, point 1 in Fig 1B) and a standard stretch on the three most distinctive repeatable features of stretch-evoked receptor potentials: the absolute voltage of the initial peak depolarisation (*E*_*p*_, 2), the hold potential during static stretch (*E*_*hold*_, 6) and the post-release hyperpolarisation (*E*_*h*_, 7). *E*_*p*_ and *E*_*hold*_ varied in proportion to *E*_*mrest*_, whilst *E*_*h*_ remains comparatively constant (Fig 2A and 2B). Additionally, we observed that, regardless of *E*_*mrest*_, the change in membrane potential to reach *E*_*p*_ (*dEm* = *E*_*p*_-*E*_*mrest*_) remained relatively constant, for a given stimulus length (Fig 2C). These relationships are very similar to those described previously in mammalian muscle spindles [7].

Additionally, muscle spindles show *E*_*p*_ values that are proportional to the amplitudes of the applied stretch stimuli [7]. We find that *dbd* neurons exhibit a similar trait. Receptor potential recordings were made for *dbd* neurons sequentially stimulated by increasing mechanical displacements of 76–92μm to the larval pelt and *E*_*p*_ was similarly proportional to the amplitudes of the stretch stimuli (Fig 2D). In conclusion, these data demonstrate that *dbd* neurons behave as stretch receptors and that they have properties similar to those of vertebrate muscle spindles.

### Modelling the receptor potential predicts that it strongly depends upon MSC activation

An *in silico* biophysical model of the receptor potentials generated in stretch-sensitive endings in response to mechanical stimuli has been described based on data obtained from crayfish stretch receptors [10]. In this model, the tensile force in the stretch receptor (modelled as a Voigt element) affects the open probability of a mechanosensory cation channel (MSC), leading to proportional depolarisation of the cell membrane. In previous work, we extended this model to include elements with characteristics of voltage-gated sodium and potassium currents (VNaC and VKC) [11]. In that extended model, the gating of these channels is entirely dependent upon MSC-mediated initial depolarisation, and they contribute to the large receptor potential response (*E*_*p*_) in the dynamic phase of the stretch. As modelled, these voltage-gated channels rapidly inactivate, leaving the MSC alone to mediate the static response phase. This model was then able to reproduce many aspects of the receptor potential from mammalian muscle spindles [11].

**Fig 3:**
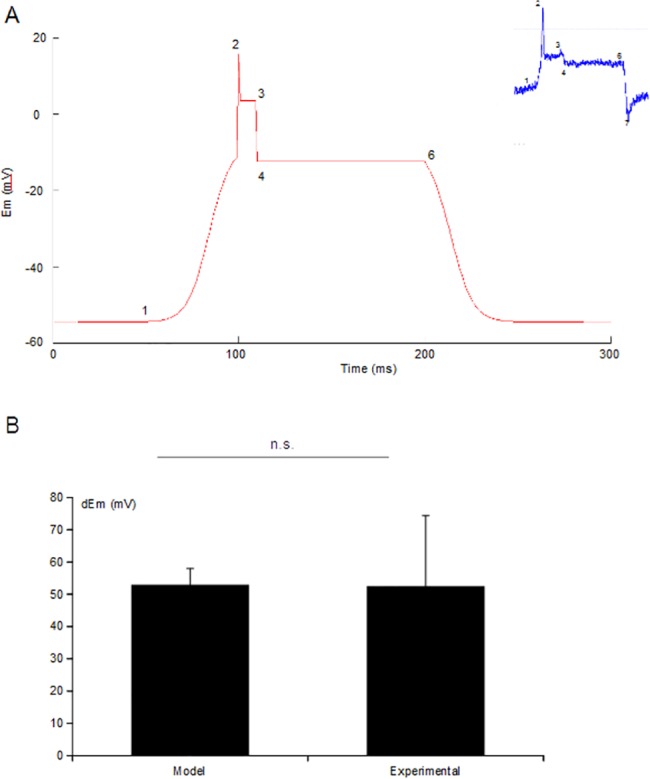
Improved in silico model of the Drosophila mechanosensory receptor potential. (A) Our *in silico* model accurately reproduces the most striking features (1–6) of the dynamic and static stretch responses seen in *dbd* neurons up to the post-release phase (inset). Modelling this latter aspect is under development. (B) In the *in silico* model, the values of *dEm* (*dEm* = *E*_*p*_-*E*_*mrest*_) accurately reflects the mean observed values of *dEm* in *dbd* neurons (Fig 2C). As might be expected for natural biological tissues the experimental data show more variability (*n* = 10, *p =* 0.5).

For the present study, we recognised that the previous model ignored any presence of calcium in the system. Thus, we refined the model further by assigning the putative VNaC activated by the mechanically gated channel a higher reversal potential (+70mV rather than +50mV). Although initially modelled based on parameters expected of a VNaC, we propose that this component could equally represent a voltage-gated Ca^2+^ current under the high-Mg^2+^ extracellular environment in our *Drosophila* system [23]. As a consequence of these adjustments, the refined model reproduces important aspects of the receptor potential of *dbd* neurons even more accurately (Fig 3A and 3B).

**Fig 4:**
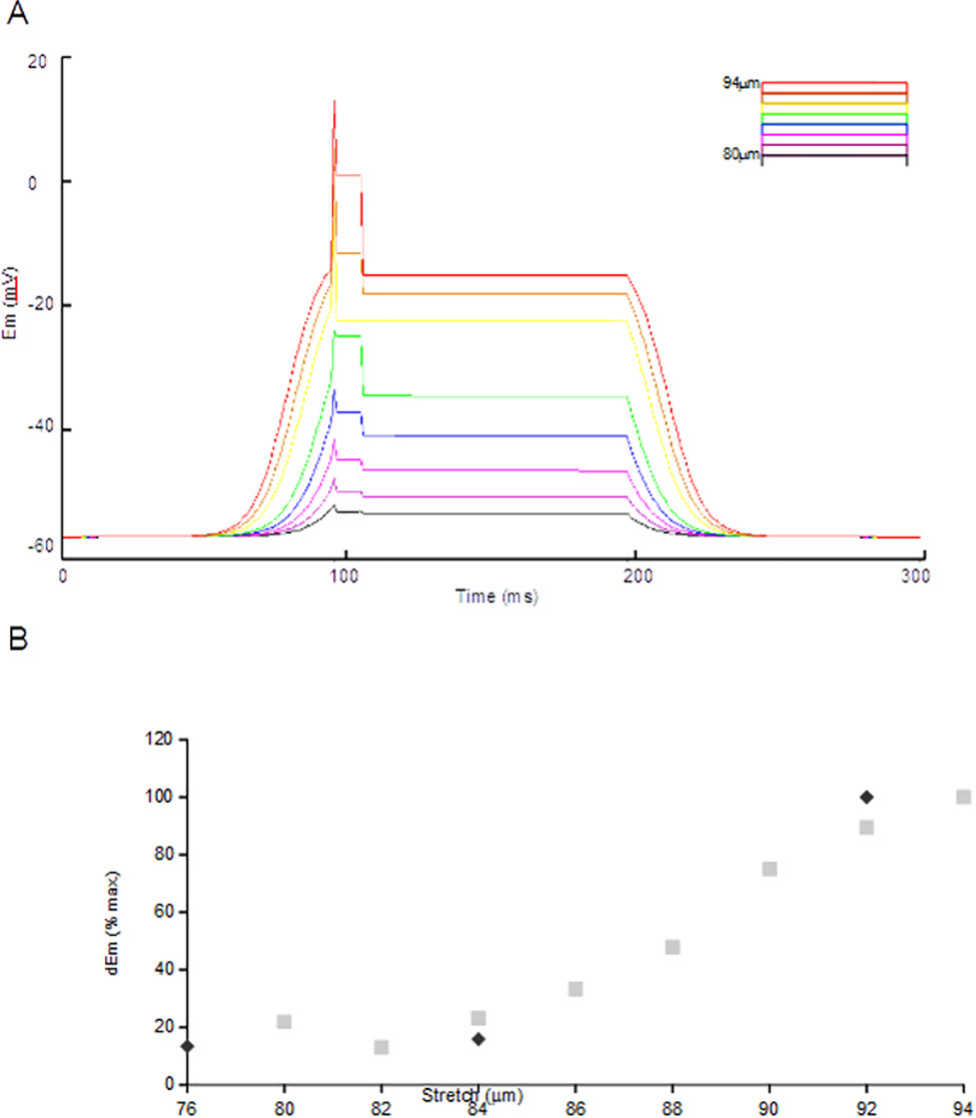
Modelling incremental inhibition of the mechanosensory sodium current proportionally inhibits all components of the receptor potential. (A) Full activation of the MSC reproduces the previously modelled behaviour (red line, as for the full response in Fig 2B). The activation term is reduced by 2μm increments, resulting in a proportional inhibition of depolarisation (sequential nested traces). The after-depolarisations (shoulder) and hold potentials are correspondingly reduced, as well. Mathematically, the model will not accept an activation value of 0, but as *MSC*_*Act*_ → 0, *dEm* → 0mV. (B) This modelled inhibition closely corresponds to stimulus-depolarisation relationship observed in our *in vivo* system. In both the *in silico* (light squares) and *in vivo* (dark diamonds–data from Fig 2D) systems, *dEm* appears to vary sigmoidally with stimulus amplitude.

Using this model, the effect of modulating the activity of the MSC was investigated by incrementally reducing the MSC activation term by the equivalent of 2μm steps. As the activity of this single channel was reduced, but leaving all other terms unaltered including the VNaC, a corresponding reduction in initial stretch-activated depolarisation was observed (Fig 4A). Furthermore, a corresponding, step-wise inhibition of the remaining phases of the modelled response was also seen, *i*.*e*., the amplitudes of the after-depolarisation (phase 3 in Fig 3A) and hold potential (4–6 in Fig 3A) were equally reduced. This is as expected since, in the model architecture, all downstream components rely upon the activation of the MSC. However, this relationship is not linear, but rather sigmoidal, with the steepest response gain over a relatively narrow range of stimuli (Fig 4B). This is of interest as it accords both with our recorded *dbd* neuron responses across a similar response range (Fig 4B), and previous observations of muscle spindles [7].

The model also predicts that the effect of stretch-stimulus modulation should be similar to progressive MSC inhibition under a constant stimulus. That is, inhibition of the MSC, modelled as a reduction in the activation term, inhibits both the stretch-evoked initial depolarisation and generation of the hold potential. This suggests that all aspects of the mechanotransduction response profile in stretch-activated endings are dependent, directly or indirectly, upon just the one component: the MSC. This prediction guided our subsequent *in vivo* experiments.

### Stretch-dependent transduction in *dbd* neurons is inhibited by NMDG, amiloride and ruthenium red

**Fig 5:**
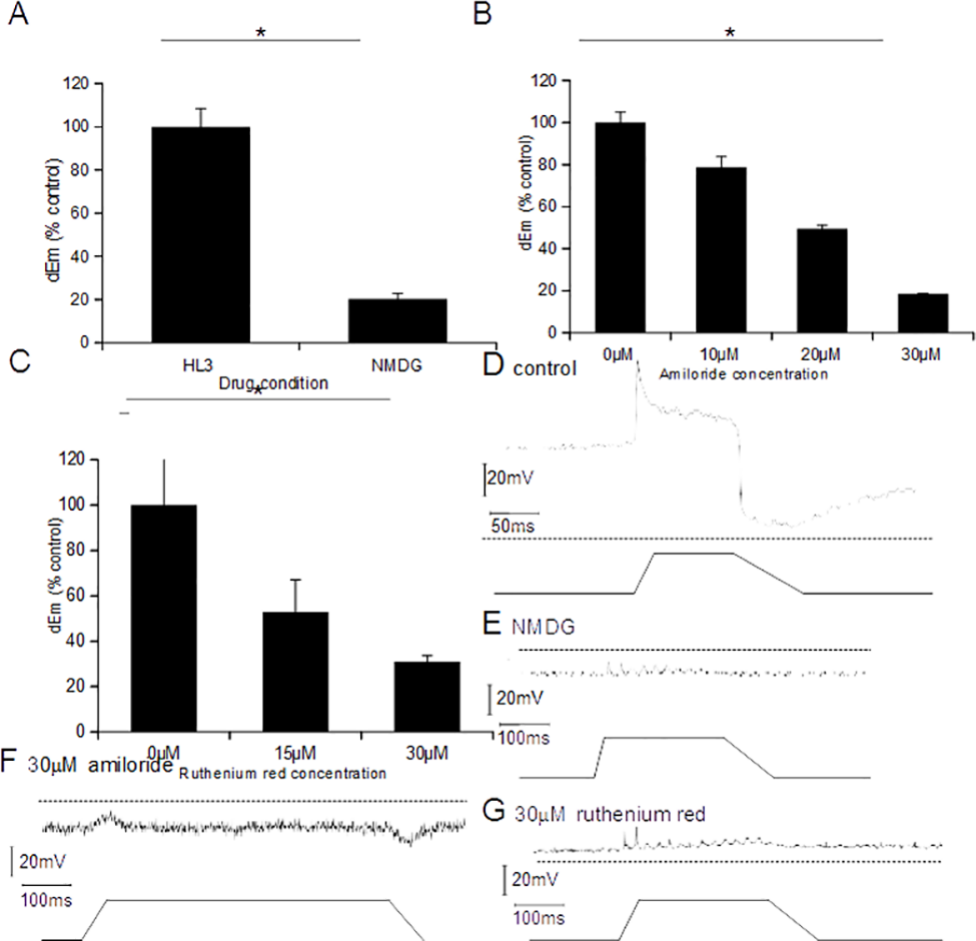
Receptor depolarisation in *dbd* neurons is inhibited by blocking a sodium-dependent MSC. (A) Replacing Na^+^ in the extracellular medium with NMDG resulted in a significant reduction in *dEm* in stretch-evoked responses by 79.8% (±2.7%, *p<*0.0001, *n =* 3). (B) Stretch-evoked depolarisation of the receptor ending in *dbd* neurons was inhibited by amiloride. The change in membrane potential (*dEm*) is normalised to the pre-drug control. Depolarisation was reduced in a dose-dependent manner (*n =* 5, *p<*0.001). (C) Ruthenium red application also reduces stretch-evoked depolarisation dose dependently (*n =* 7, *p<*0.0001). (D-G) Representative traces for control (D), NMDG (E), amiloride (F) and ruthenium red (G).

Our current understanding of mechanotransduction in muscle spindles assumes that the primary MSC in stretch-activated neurons should be a mechanosensory sodium channel [7, 10]. Therefore, in our *dbd* neuron system, we tested the effect of replacing Na^+^ in the extracellular saline with NMDG, to block sodium conductance. Stretch-evoked recordings were initially made from *dbd* neurons in standard HL3 saline containing 80mM Na^+^. HL3 with NMDG-Cl substituted for Na^+^ was then washed on. As predicted, stretch-evoked depolarisation was substantially reduced by exchanging extracellular Na^+^ for NMDG (Fig 5A and 5E). Moreover, in addition to the reduced *E*_*p*_, the rest of the stretch-evoked response was absent, as was predicted by the *in silico* model. This shows that, as in mammalian spindles [7], Na^+^ is an important component of the *dbd* receptor potential. However, although Na^+^ was completely replaced with NMDG, the receptor potential was not entirely absent, with ~20% remaining. This is consistent with a similar contribution by additional ions to the stretch-activated receptor potential, as reported in mammals [7]. In mammalian muscle spindles, the residual potential was due to a stretch-activated Ca^2+^ current.

We next attempted to identify the MSC involved. A number of known channels could perform the role of MSC highlighted by our model. Mammalian muscle spindles are sensitive to the broad-spectrum MSC inhibitor amiloride and its analogues [24], which also blocks many other MSCs [25, 26]. Therefore, we investigated whether it also inhibited the response of *dbd* neurons by recording stretch-evoked receptor potentials in increasing concentrations of amiloride. Amiloride reduced the receptor potential difference *dEm* in a dose-dependent manner, producing an ~80% inhibition at 30μM (n = 5, *p* = 0.001) (Fig 5B and 5F). This is consistent with previous results in mammalian muscle spindles, where 100μM amiloride blocked responses by ~75% [24].

While there is no evidence of ENaC expression in *dbd* neurons [27, 28], TRP and Piezo family members are also possibilities for MSCs in *Drosophila*. Two such proteins are expressed in *dbd* neurons: TRPA1 [13] and DmPiezo [14]. Mammalian TRPA1 and Piezo are both blocked by ruthenium red [29, 30]. Therefore, we subsequently tested the effect of ruthenium red on stretch-evoked *dEm* responses. In *Piezo*-transfected HEK239T cells, 30μM ruthenium red reduces Piezo-mediated cation currents, by ~80% [31]. Consistent with this, we found that exposure to 15μM and 30μM ruthenium red produced a strong, dose-dependent inhibition of the stretch-evoked depolarisation in *dbd* neurons (Fig 5Cand 5G). These results suggested that one or both of these channels could indeed be mediating *dbd* neuron responses. Distinguishing between these possibilities was pursued using genetic approaches.

### *DmPiezo*, not TRPA1, is the primary mechanosensory channel in *dbd* neurons

**Fig 6:**
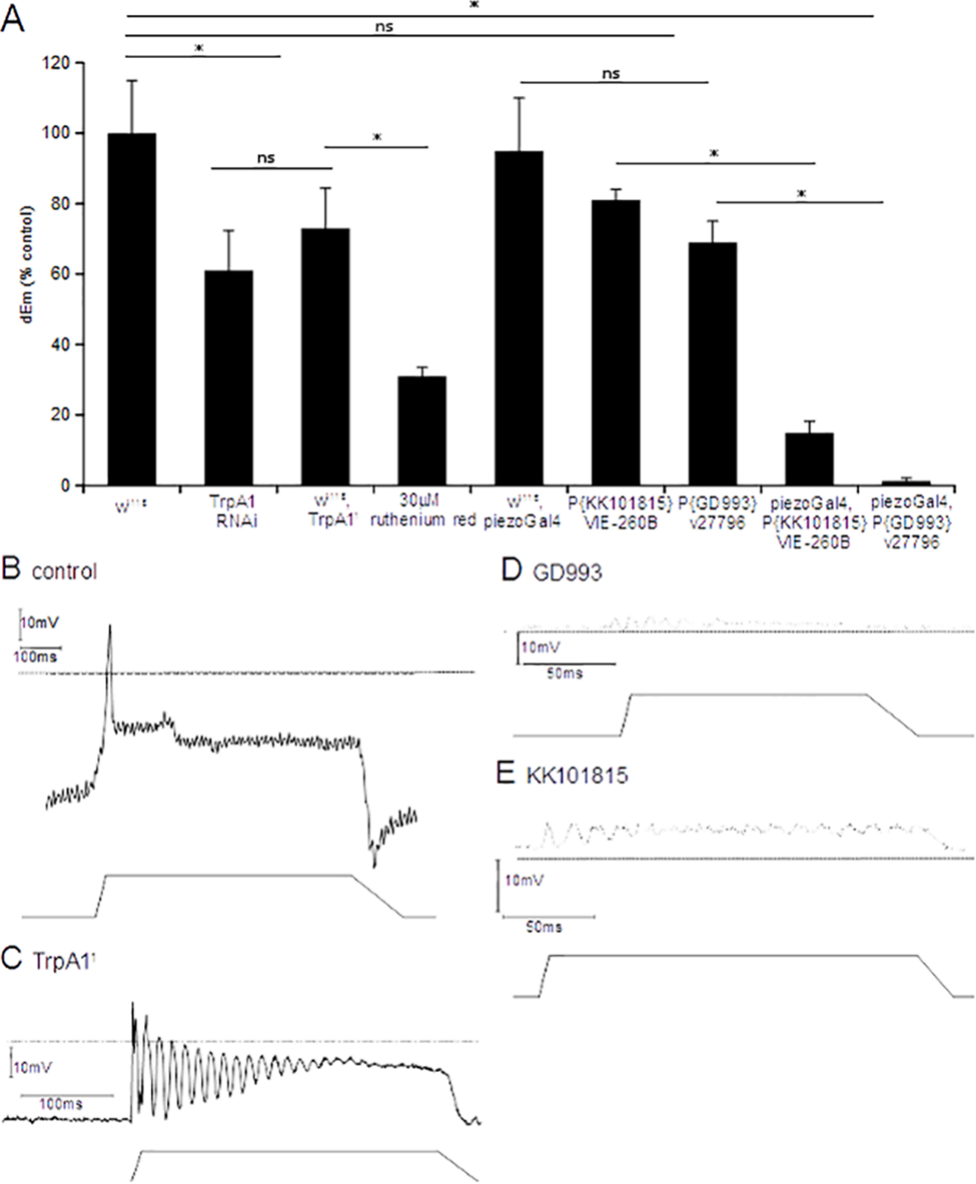
Loss of TRPA1 and dmPiezo function have small and large effects respectively on response to stretch. (A) Stretch-evoked depolarisation was recorded in response to maximal stretch stimuli in *dbd* neurons of wild-type (*w* ^*1118*^), *TrpA1* ^*1*^ mutants or RNAi knockdown larvae. Data were compared to the previously-measured effect on *w* ^*1118*^ treated with 30μM ruthenium red (Fig 5). Loss of TRPA1 inhibited stretch-evoked depolarisation <20% (*n =* 3, *p<*0.0001), whereas the inhibition by ruthenium red is at least 3x more profound (*n =* 7, *p<*0.0001). RNAi knock-down of TRPA1 produced a similar partial blockade of stretch-evoked depolarisation (*dEm* = 68% control, *n* = 3, *p<*0.0001). However, when *DmPiezo* expression is reduced via RNAi knock-down, the receptor potential is almost completely abolished *(E*_*p*_<5mV) compared to corresponding controls (GD993 –*dEm* = 1.3% controls, *n* = 3, KK101815 –*dEm* = 14.9% controls, *n* = 4, *p<*0.0001). (B-E) Representative traces for control (B), *TrpA1* ^*1*^ mutant (C), *dmPiezo* knockdown line GD993 (D) and *dmPiezo* knockdown line KK101815 (E).

To examine the role of TRPA1, both knock-down of TRPA1 via RNAi and larvae homozygous for the genetic null *TrpA1* ^*1*^ mutation [32] were tested. RNAi knock down of TRPA1 produced a small but significant reduction in receptor potential (*dEm*≈-20%, *p*<0.0001; Fig 6A). When compared to wild-type, *dbd* neurons in genetic null *TrpA1* ^*1*^ larval pelts exhibited a very similar partial depolarisation block (Fig 6A and 6C). Together, these indicate that TRPA1 makes a distinct but small contribution to the stretch-response of these neurons. It is clearly insufficient to explain the effect of ruthenium red.

Given the small effect of TRPA1 deletion, the large effect of ruthenium red suggested that *DmPiezo* is the predominant MSC in *dbd* neurons. To investigate this hypothesis, we examined the effect of specific RNAi knock-down of *DmPiezo*, the only Piezo family member in *Drosophila*. RNA interference was tested for two independent *Piezo-*RNAi knock-down lines, driven in *dbd* neurons by *Piezo-Gal4*, which is active in *dbd* neurons (*Piezo-Gal4*, *UAS-PiezoRNAi* larvae). In contrast to the modest effect of the TRPA1 manipulations, when *DmPiezo* expression is reduced via RNAi knock-down, a substantial reduction of *dbd* neuron receptor potential was observed in both *DmPiezo* knock-down lines (Fig 6A, 6D and 6E), with *dEm* being below 5mV for maximal stimuli, compared to an amplitude of 56±0.72mV for wild-type neurons. This indicates that DmPiezo is the primary MSC responsible for the stretch-evoked response in *dbd* neurons. These data indicate that the inhibition by both amiloride and ruthenium red, described above, are likely to be mediated via their interactions with DmPiezo.

### Mammalian muscle spindle responses exhibit similar pharmacology to *dbd* neurons

In muscle spindles, inhibition of stretch-evoked firing by amiloride and its analogues, antibody immunoreactivity in cryosections and Western blots have shown the presence of ENaC and ASIC2 channels [24]. Being highly Na^+^ selective, these channels presumably account for the large Na^+^ contribution to the mammalian receptor [7]. However, there is also a 20% contribution from a Ca^2+^ current to the potential, which may be mediated by TRP or Piezo channels. While there have been no previous reports of TRPs or Piezo 1 or 2 expression specifically in spindle terminals, there is evidence that both nociceptive and non-nociceptive DRG neuronal cell bodies express Piezos [29], as well as light-touch sensitive Merkel cells and their associated primary afferent terminals [33, 34]. We therefore asked whether these proteins contribute to the stretch-evoked responses in muscle spindle sensory endings, as they do in *dbd* neurons.

**Fig 7:**
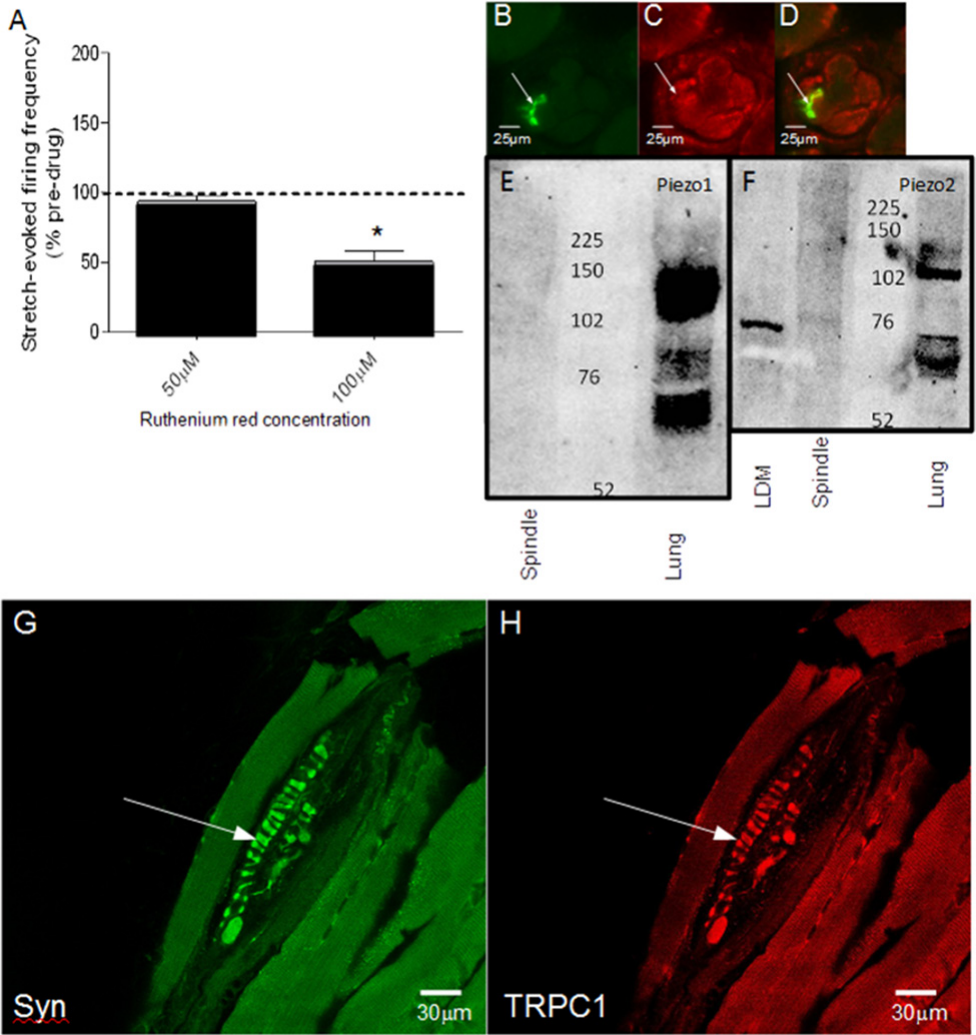
Ruthenium red inhibits stretch-evoked firing of muscle spindle afferents, but not via Piezo. (A) Ruthenium red substantially decreases stretch-evoked afferent firing at 100μM (*p*<0.001, *n =* 6), but not at 50μM *(n* = 7). (B–D) Cryosection of medial portions of deep masseter muscle containing muscle spindles. Section immunolabelled for synaptophysin (B) and Piezo2 (C) showing prominent synaptophysin labelling in spindle sensory terminals (arrows), but nothing above background was seen for Piezo2. (D) Merge image of the above. (E,F) Western blots for the expression of Piezo1 (E) or Piezo2 (F). A very similar banding pattern was seen with the other Piezo2 antibody (AbCam FAM38B). In all three cases, no expression could be detected in muscle spindle homogenates, with two being illustrated here. (G,H) Rat muscle spindle annulospiral primary afferent endings (arrows), identified by synaptophysin-positive labelling of their synaptic-like vesicles (green), show high TRPC1 immunoreactivity (red).

As direct recording of receptor potentials in muscle spindles is technically challenging, stretch-evoked afferent firing rate was used as a proxy measurement of receptor potential generation, as the two are known to be causally and directly related [12]. Just as in *dbd* neurons, at 100μM ruthenium red caused a significant decrease (-50.2%) in stretch-evoked afferent firing (*p* = 0.001 paired t-test, *n* = 6) (Fig 7A). Since ruthenium red does not inhibit ENaCs and ASICs [35] this suggests that TRP and/or Piezo channels also contribute to the receptor potential. However, unlike in *dbd* neurons, we could find no evidence for Piezo1 or Piezo2 in spindle afferent terminals, either by immunohistochemistry (Fig 7B–7D), Western blotting (Fig 7E and 7F) or mass spectrometry, although we were able to confirm the strong *Piezo1* and *2* expression in lung. There was also modest expression of Piezo2 in spindle-free regions of the skeletal muscle (lateral deep masseter), consistent with reports of expression in these tissues previously [29]. In contrast, we did detect TRPC1 and TRPV3 by both immunoreactivity (Fig 7G and 7H) and mass spectrometry (amino acids 6–16 ‘KEMAPLMGKRT’ of mouse TRPV3, MASCOT score 37, 1 match, E-value 0.043), which suggests that it is inhibition of TRP channels that reduces stretch-evoked firing in the presence of ruthenium red.

## Discussion

This study establishes the *Drosophila dbd* neuron as a useful, accessible and tractable *in vivo* model for studying the phenomenon of mechanotransduction in stretch receptor neurons. It also shows the utility of an *in silico* model for identifying components of a mechanosensitive system. Whilst earlier studies have utilised electrophysiology of *Drosophila* neurons of other sensory modalities [22, 16] we are unaware of any study utilising this approach in *Drosophila* to test *in vivo* responses to physiologically relevant mechanical stimuli. In combination with the predictive capacity of mathematical modelling, this promises to be a very powerful tool for dissecting the process of mechanotransduction and identifying transducer proteins that are activated by mechanical stimuli in the physiological range. In this study, the contribution of a Piezo protein to a innocuous stretch-activated cellular response in fully differentiated neurons has been directly demonstrated.

Members of three channel families are currently strongly implicated in mechanotransduction: DEG/ENaCs, TRPs, and more recently Piezo proteins [29, 36, 33]. Of these, Piezo protein functions are the least well characterised. Piezo proteins can gate mechanically sensitive currents when expressed in cultured cells, but their *in vivo* functions are less well known. Recent studies showed that Piezo2b is expressed in zebrafish somatosensory Rohon-Beard cells and is required for behavioural response to light touch [37], while Piezo2 in mouse is required for touch sensation [34]. In contrast, *Drosophila* DmPiezo is required in sensory neurons for behavioural responses to noxious touch but not innocuous touch [32]. To these studies, our findings now demonstrate a role for DmPiezo in a innocuous stretch-mediated receptor response, with direct evidence for an *in vivo* electrophysiological requirement for DmPiezo.

The role of Ca^2+^ in the receptor response remains to be explored further. NMDG substitution results in a ~20% residual current, suggesting a contribution of Ca^2+^ to the receptor potential. Our data show that DmPiezo plays the major role in producing the receptor potential, but it is a non-selective cation channel [38] and likely conducts both Na^+^ and Ca^+^ in *dbd* neurons. However, as Ca^2+^ is the main permeant ion for TRPA1 channels, the residual current may reflect TRPA1’s contribution. Suggestive of this is the observation that the reduction in *E*_*p*_ upon TRPA1 knock-down is quantitatively similar to the current remaining when Na^+^ is removed from the extracellular saline by NMDG substitution. Thus, there seems to be a ~20% contribution of Ca^2+^ to the receptor potential. Conversely, it may be that TRPA1’s involvement is indirect, as it can both modulate and co-precipitate with Piezo [39], although in the latter study the modulatory interaction was negative. The essentially complete block of mechanosensory response in the most effective of the two *DmPiezo* RNAi strains argues more in favour of an interactive regulation between the two channels rather than an independent contribution of TRPA1 to the receptor potential (Fig 7).

A putative sensory transduction role for TRPA1 in *dbd* neurons had been previously identified, but this was specifically in a thermoreceptive capacity [13]. Further examination of this potentially bimodal sensory role of TRPA1 in the *dbd* receptor, and how it may interact with DmPiezo may provide useful insights into primary sensory transduction pathways. For example, Ca^2+^ influx through mammalian TRPA1 has a strong role in activating TRPV1 channels in nociceptive neurons [40]. Our modelling has indicated that the immediate downstream component of a stretch-transducing MSC may be a *voltage-gated* channel conducting either Na^+^ or Ca^2+^. It is possible that TRPA1 may fulfil this role, but it has not been reported to be voltage sensitive, this may indicate the involvement of yet another channel.

Amiloride produced a profound inhibition of stretch-evoked responses at only 30μM. While it is possible that this inhibition is secondary to blockade of TRPA1, this seems unlikely as TRPA1 knockdown has only a modest effect. Thus, Piezo channels seem to be directly sensitive to amiloride and, if so, this is the first such report.

It is interesting to note that the quantitative contribution made by Ca^2+^ to the stretch-activated receptor potential in both systems, the *dbd* neurons and mammalian muscle spindles, is similar at ~20% [7]. While there have been no reports of specifically TRPA1 in spindles, the expression of TRPC1 and TRPV3 we have uncovered in muscle spindle afferent terminals could equally be the basis of such a Ca^2+^ current. They may also be the source of the Ca^2+^ influx in spindle terminals responsible for the Ca^2+^-mediated activation of synaptic-like vesicle recycling in these endings [18].

The similarity of the overall profile of the stretch-evoked receptor potential in *dbd* neurons and mammalian muscle spindles is striking. Our *in silico* model provides an electrophysiological mechanism to describe these stretch receptor potential behaviours in terms of the Na^+^, K^+^ and Ca^2+^ currents thought to be involved, based on previous studies in invertebrate and mammalian systems [41, crayfish stretch receptor; 7, muscle spindles]. However, it now appears that the specifics of the molecular components responsible for these currents differ between these two systems. While mammalian Piezo2 is expressed in some DRG neurons [29, 36, 33], including light touch receptors [34], we have so far found no evidence for Piezo expression in muscle spindle terminals. Instead, immunocytochemistry, expression and pharmacological evidence suggests that ENaCs play the key role of carrying the Na^+^ current in spindles [24]. The overall complex profile, therefore, seems of great importance whilst the details of the channels responsible for carrying the major, Na^+^- and Ca^2+^-dependent components of the receptor potential may vary.

## References

[1] Delmas P, Hao J, Rodat-Despoix L. Molecular mechanisms of mechanotransduction in mammalian sensory neurons. Nat Rev Neurosci. 2011; 140(12): 139–53. 10.1038/nrn2993 21304548

[2] Proske U, Gandevia SC. The proprioceptive senses: their roles in signalling body shape, body position and movement, and muscle force. Physiol Rev. 2012; 92: 1651–97. 10.1152/physrev.00048.2011 23073629

[3] Finlayson LH, Loewenstein O. The structure and function of abdominal stretch receptors in insects. Proc R Soc Lond B Biol Sci. 1958; 148(933): 433–49. 13542636 10.1098/rspb.1958.0037

[4] Tamarkin DA, Levine RB. Synaptic interactions between a muscle-associated proprioceptor and body wall muscle motor neurons in larval and adult *Manduca sexta* . J Neurophysiol. 1996; 76(3): 1597–610. 8890279 10.1152/jn.1996.76.3.1597

[5] Simon MA, Trimmer BA. Movement encoding by a stretch receptor in the soft-bodied caterpillar, *Manduca sexta* . J Exp Biol. 2009; 212: 1021–31. 10.1242/jeb.023507 19282499

[6] Schrader S, Merritt DJ. Dorsal longitudinal stretch receptor of *Drosophila melanogaster* larva—fine structure and maturation. Arthropod Struct Dev. 2007; 36(2): 157–69. 18089096 10.1016/j.asd.2006.08.014

[7] Hunt CC, Wilkinson RS, Fukami Y. Ionic basis of the receptor potential in primary endings of mammalian muscle spindles. J Gen Physiol. 1978; 71: 683–98. 149839 10.1085/jgp.71.6.683 PMC2215112

[8] Ottoson D, Swerup C. Ionic dependence of early adaptation in the crustacean stretch receptor. Brain Res. 1985; 336: 1–8. 4005570 10.1016/0006-8993(85)90409-3

[9] Rydqvist B, Purali N. Transducer properties of the rapidly adapting stretch receptor neurone in the crayfish (*Pacifastacus leniusculus*). J Physiol. 1993; 469: 193–211. 8271197 10.1113/jphysiol.1993.sp019811 PMC1143868

[10] Swerup C, Rydqvist B. A mathematical model of the crustacean stretch receptor neuron. Biomechanics of the receptor muscle, mechanosensitive ion channels, and macrotransducer properties. J Neurophysiol. 1996; 76: 2211–20. 8899596 10.1152/jn.1996.76.4.2211

[11] Suslak TJ, Armstrong JD, Jarman AP. A general mathematical model of transduction events in mechano-sensory stretch receptors. Network. 2011; 22(1–4): 133–42. 10.3109/0954898X.2011.638967 22149673

[12] Bewick GS, Banks RW. Mechanotransduction in the muscle spindle. Eur J Physiol. 2014; 1–6. 10.1007/s00424-014-1536-9 PMC4281366 24888691

[13] Shen WL, Kwon Y, Adegbola AA, Luo J, Chess A, Montell C. Function of rhodopsin in temperature discrimination in *Drosophila* . Science. 2011; 331: 1333–6. 10.1126/science.1198904 21393546

[14] Kim SE, Coste B, Chadha A, Cook B, Patapoutian A. The role of *Drosophila* Piezo in mechanical nociception. Nature. 2012; 483: 209–13. 10.1038/nature10801 22343891 PMC3297676

[15] Stewart BA, Atwood HL, Renger JJ, Wang J, Wu CF. Improved stability of *Drosophila* larval neuromuscular preparations in haemolymph-like physiological solutions. J Comp Physiol (A). 1994; 175(2): 179–91. 8071894 10.1007/BF00215114

[16] Nair A, Bate M, Pulver SR Characterization of voltage-gated ionic currents in a peripheral sensory neuron in larval *Drosophila* . BMC Res Notes. 2010; 3: 154–61. 10.1186/1756-0500-3-154 20525165 PMC2893198

[17] Jan LY, Jan YN. L-glutamate as an excitatory transmitter at the *Drosophila* larval neuromuscular junction. J. Physiol. 1976; 262: 215–236. 186587 10.1113/jphysiol.1976.sp011593 PMC1307638

[18] Bewick GS, Reid B, Richardson C, Banks RW. Autogenic modulation of mechanoreceptor excitability by glutamate release from synaptic-like vesicles: evidence from the rat muscle spindle primary sensory ending. J Physiol. 2005; 562: 381–94. 15528245 10.1113/jphysiol.2004.074799 PMC1665510

[19] Liley AW. An investigation of spontaneous activity at the neuromuscular junction of the rat. J Physiol. 1956; 132: 650–66. 13332600 10.1113/jphysiol.1956.sp005555 PMC1363576

[20] Lennartson B. Number and distribution of muscle spindles in the masticatory muscles of the rat. J Anat, 1988; 130: 279–88. PMC1233132 6447135

[21] Laemmli UK. Cleavage of structural proteins during the assembly of the head of bacteriophage T4. Nature. 1970; 227: 680–5. 5432063 10.1038/227680a0

[22] Baines RA, Bate M. Electrophysiological development of central neurons in the *Drosophila* embryo. J Neurosci. 1998; 18(12): 4673–83. 9614242 10.1523/JNEUROSCI.18-12-04673.1998 PMC6792699

[23] Campbell DL, Giles WR, Hume JR, Noble D, Shibata EF. Reversal potential of the calcium current in bull-frog atrial myocytes. J Physiol. 1988; 403: 267–86. 2855342 10.1113/jphysiol.1988.sp017249 PMC1190713

[24] Simon A, Shenton F, Hunter I, Banks RW, Bewick GS. Amiloride-sensitive channels are a major contributor to mechanotransduction in mammalian muscle spindles. J Physiol. 2010; 588: 171–85. 10.1113/jphysiol.2009.182683 19917568 PMC2821557

[25] Lane JW, McBride DW Jr, Hamill OP. Amiloride block of the mechanosensitive cation channel in *Xenopus* oocytes. J Physio. 1991; 441: 347–66. 10.1113/jphysiol.1991.sp018755 PMC1180202 1816379

[26] Carr MJ, Gover TD, Weinreich D, Undem BJ. Inhibition of mechanical activation of guinea-pig airway afferent neurons by amiloride analogues. Br J Pharmacol. 2001; 133: 1255–62. 11498511 10.1038/sj.bjp.0704197 PMC1621149

[27] Adams CM, Anderson MG, Motto DG, Price MP, Johnson WA, Welsh MJ. *Ripped Pocket* and *Pickpocket*, novel *Drosophila* DEG/ENaC subunits expressed in early development and in mechanosensory neurons. J Cell Biol. 1998; 140: 143–52. 9425162 10.1083/jcb.140.1.143 PMC2132602

[28] Liu L, Johnson WA, Welsh MJ. *Drosophila* DEG/ENaC pickpocket genes are expressed in the tracheal system, where they may be involved in liquid clearance. PNAS. 2003; 100(4): 2128–33. 12571352 10.1073/pnas.252785099 PMC149970

[29] Coste B, Mathur J, Schmidt M, Earley TJ, Ranade S, Petrus MJ, et al Piezo1 and Piezo2 are essential components of distinct mechanically activated cation channels. Science. 2010; 330: 55–60. 10.1126/science.1193270 20813920 PMC3062430

[30] Banke TG. The dilated TRPA1 channel pore state is blocked by amiloride and analogues. Brain Res. 2011; 1381: 21–30. 10.1016/j.brainres.2011.01.021 21241666

[31] Coste B, Xiao B, Santos JS, Syeda R, Grandl J, Spencer KS, et al Piezo proteins are pore-forming subunits of mechanically activated channels. Nature. 2012; 483: 176–83. 10.1038/nature10812 22343900 PMC3297710

[32] Kim SH, Lee Y, Akitakea B, Woodward OM, Guggino WB, Montell C. *Drosophila* TRPA1 channel mediates chemical avoidance in gustatory receptor neurons. PNAS. 2010; 107(18): 8440–5. 10.1073/pnas.1001425107 20404155 PMC2889570

[33] Woo S-H, Ranade S, Weyer AD, Dubin AE, Baba Y, Qiu Z, et al Piezo2 is required for Merkel-cell mechanotransduction. Nature. 2014; 509: 622–626. 10.1038/nature13251 24717433 PMC4039622

[34] Ranade SS, Woo S-H, Dubin AE, Moshourab RA, Wetzel C, Petrus M, et al Piezo2 is the major transducer of mechanical forces for touch sensation in mice. Nature. 2014; 516: 121–125. 10.1038/nature13980 25471886 PMC4380172

[35] Hu J, Lewin GR. Mechanosensitive currents in the neurites of cultured mouse sensory neurones. J Physiol. 2006; 577: 815–828. 17038434 10.1113/jphysiol.2006.117648 PMC1804210

[36] Maksimovic S, Nakatani M, Baba Y, Nelson AM, Marshall KL, Wellnitz SA, et al Epidermal Merkel cells are mechanosensory cells that tune mammalian touch receptors. Nature. 2014; 509: 617–621. 10.1038/nature13250 24717432 PMC4097312

[37] Faucherre A, Nargeot J, Mangoni ME, Jopling C. piezo2b regulates vertebrate light touch response. J Neurosci. 2013; 33(43): 17089–94. 10.1523/JNEUROSCI.0522-13.2013 24155313 PMC6618434

[38] Bobkov YV, Corey EA, Ache BW. The pore properties of human nociceptor channel TRPA1 evaluated in single channel recordings. Biochem Biophys Acta. 2011; 1808(4): 1120–8. 10.1016/j.bbamem.2010.12.024 21195050 PMC3062711

[39] Peyronnet R, Martins JR, Duprat F, Demolombe S, Arhatte M, Jodar M, et al Piezo1-dependent stretch-activated channels are inhibited by polycystin-2 in renal tubular epithelial cells. EMBO Rep. 2013; 14: 1143–8. 10.1038/embor.2013.170 24157948 PMC3981085

[40] Staruschenko A, Jeske NA, Akopian AN. Contribution of TRPV1-TRPA1 interaction to the single channel properties of the TRPA1 channel. J Biol Chem. 2010; 285(20): 15167–77. 10.1074/jbc.M110.106153 20231274 PMC2865321

[41] Ottoson D. Swerup C. Studies on the role of calcium in adaptation of the crustacean stretch receptor. Effects of intracellular injection of calcium, EGTA and TEA. Brain Res. 1982; 244: 337–341. 6288188 10.1016/0006-8993(82)90093-2

